# Evaluating a Novel 3D-Printed Resin for Dental Restorations: Fracture Resistance of Restorations Fabricated by Digital Press Stereolithography

**DOI:** 10.3390/polym17172322

**Published:** 2025-08-27

**Authors:** Cristian Abad-Coronel, Cinthya Freire Bonilla, Sebastián Vidal, Fabián Rosero, Carolina Encalada Abad, Nancy Mena Córdova, César A. Paltán, Jorge I. Fajardo, Paulina Aliaga

**Affiliations:** 1 CAD/CAM and Digital Dentistry Research Group, Faculty of Dentistry, Universidad de Cuenca, Cuenca 010107, Ecuador; sebastian.vidal@ucuenca.edu.ec (S.V.); caencala@ucm.es (C.E.A.); 2 Department of Prosthodontics, Faculty of Dentistry, Universidad San Francisco de Quito, Quito 170901, Ecuador; cfreireb1@estud.usfq.edu.ec (C.F.B.); nmena@usfq.edu.ec (N.M.C.); paliaga@usfq.edu.ec (P.A.); 3 Private Practice, Quito 170517, Ecuador; orthodiagnostic3d@gmail.com; 4 Mechanical Enginnering New Materials and Transformation Processes Research Group (GiMaT), Universidad Politécnica Salesiana, Cuenca 170517, Ecuador; cpaltan@ups.edu.ec (C.A.P.); jfajardo@ups.edu.ec (J.I.F.)

**Keywords:** CAD/CAM materials, biomechanical testing, 3D printing, digital press stereolithography (DPS), indirect restoration, dental polymers

## Abstract

An in vitro study evaluated the fracture resistance of four CAD/CAM restorative materials: lithium disilicate ceramic (IPS e.max CAD, EM), hybrid ceramic (Vita Enamic, VE), a polymer-based composite (Cerasmart, CS), and a novel 3D-printed resin (Ceramic Crown, CC) fabricated using digital press stereolithography (DPS) technology. Standardized full-coverage crowns were designed and manufactured for each material. All specimens underwent thermocycling and fracture testing using a universal testing machine. EM exhibited the highest fracture resistance (mean: 440.49 N), while VE showed the lowest (173.82 N). CS (265.49 N) and CC (306.76 N) presented intermediate values without statistically significant differences between them. Stereomicroscopic analysis revealed differences in fracture patterns, with IPS e.max CAD showing smooth, brittle fractures, while hybrid and polymer-based materials exhibited tortuous fracture surfaces. These results suggest that DPS technology achieves mechanical performance for Ceramic Crown comparable to that of milled polymer-based composites, while offering production advantages in terms of time efficiency. As one of the first studies to evaluate Ceramic Crown and DPS technology, these findings provide initial insights into their mechanical behavior. However, further studies are required to validate their clinical performance before widespread use can be recommended.

## 1. Introduction

Computer-aided design and computer-aided manufacturing (CAD/CAM) technologies have transformed dentistry since the 1980s [1]. CAD/CAM technology offers several advantages over conventional techniques, including improved predictability, greater reproducibility, automation of processes, and faster and more fluid storage and exchange of information [2]. There are two main workflows for obtaining restorations using CAD/CAM systems: subtractive and additive manufacturing. Current evidence indicates that 3D printing systems surpass milling systems in precision when fabricating prostheses, as they enable the production of complex geometries such as walls with concave cavities, which are difficult to achieve through subtractive techniques. Moreover, additive manufacturing minimizes material waste observed with the machining of bulky blocks and discs [3].

The advancement of digital dentistry, in addition to fostering the development of new technologies, is directly linked to the progress of ceramic, polymeric, and hybrid dental materials [4]. Among these, feldspathic ceramic with lithium disilicate is one of the most widely accepted materials due to its aesthetic properties and high load-bearing capacity in terms of flexural strength. According to the 2023 Glossary of Prosthodontic Terms (GPT), hybrid material is defined as “a porous ceramic network infiltrated by a polymer” [5]; it exhibits high mechanical strength, elasticity, and aesthetics in minimally invasive preparations [6]. Polymeric materials, on the other hand, have lower stiffness but demonstrate superior wear resistance in partial restorations. They are distinguished by their favorable biomechanical behavior in minimally invasive preparations, where the preservation of dental tissue and low antagonist abrasion are required [7,8].

A restorative material must fulfill basic requirements such as a natural aesthetic appearance, adequate marginal adaptation, and sufficient fracture resistance. Fracture resistance is defined as the maximum stress a material can withstand before failure, allowing for the assessment of the durability and performance of indirect restorations under functional and parafunctional conditions [9]. Fracture resistance is directly related to the dental preparation technique, which today seeks to preserve as much remaining structure as possible, thus demanding superior clinical performance from restorative materials [4]. Furthermore, analyzing fracture patterns provides detailed information regarding severity, distribution, and morphology, which are key aspects in assessing the possibility of structural recovery. This analysis facilitates the selection of dental materials and restorative techniques that promote long-term durability and clinical success [10].

To evaluate the mechanical behavior of dental materials, various tests are employed, including the three-point bending test, accelerated fatigue test, cyclic loading test, and static loading test. Each method allows for the analysis of specific properties critical to material selection for indirect restorations. The three-point bending test is ideal for evaluating homogeneous and brittle materials such as polymers and ceramics, as it applies a transverse load at a specific point to precisely identify the fracture limit [11]. The accelerated fatigue test evaluates a material’s resistance to repetitive loads applied over a short period, leading to failure due to fatigue phenomena [12]. The cyclic loading test subjects materials to recurrent stresses over time, allowing for the study of their response to repeated mechanical forces. Finally, the static loading test assesses the strength and deformation of a material under an axial load that may be constant or progressive until failure. This test can be performed using a hydraulic press or universal testing machine, with results quantified by the force applied until deformation. Fatigue induced by these tests is key for selecting dental materials, as each material reacts individually to cyclic stresses or deformations and must be evaluated comprehensively. Therefore, the application of cyclic and static loading tests is crucial to assessing the fracture resistance of materials, providing a more precise understanding of the structural integrity of indirect restorations [13].

Several factors inherent to dental materials determine their fracture resistance, including physical factors (such as time and corrosion), patient-related factors (occlusion, quantity and quality of remaining structure, dentin’s elastic modulus, proximity to the pulp), and operator-related factors (tooth preparation, cementation protocol, and choice of restorative material) [14]. Significant differences have been reported in the fracture resistance of hybrid and ceramic materials fabricated via subtractive techniques. For instance, studies have shown variable values for VITA Enamic (VE) (1978.71 N), Cerasmart (CS) (2578.99 N), and IPS e.max CAD (EM) (2781.51 N). These differences are primarily attributed to the distinct microstructures of each material, which influence their mechanical behavior under load. Factors such as the type and distribution of ceramic fillers, polymer matrix composition, and degree of cross-linking can markedly affect fracture resistance [15]. The study conducted by Suksawat [16] reported similar values between VE (797.32 N), EM (676.62 N), and CS (761.38 N). Therefore, it is justified to evaluate the differences in fracture resistance of indirect restorations fabricated from 3D-printed materials versus those produced using subtractive techniques.

The hybrid material VE features a dual ceramic–polymer matrix, allowing for rapid CAD/CAM fabrication with a manually polished surface that requires no post-processing firing. According to the manufacturer, its mechanical characteristics include a flexural strength of 150–160 MPa, an elastic modulus of 30 GPa, and a hardness of 2.5 GPa [17]. EM, in contrast to other ceramics, undergoes a simplified crystallization process lasting 20–31 min, which results in microstructural transformation through controlled lithium disilicate crystal growth. Its reported properties include a biaxial flexural strength of 360 MPa, elastic modulus of 95 GPa, and Vickers hardness of 5800 MPa [18]. The polymeric material CS demonstrates durable marginal sealing, a strong and balanced surface that reduces antagonist tooth wear, long-lasting gloss, milling efficiency, polishing capability, and ease of characterization. According to the manufacturer, its mechanical properties include a flexural strength of 231 MPa and a fracture toughness of 2.2 N/cm. These values fall within or above the minimum thresholds established by ISO 6872:2024 for polymer-based restorative materials intended for permanent single-unit crowns, which require a minimum flexural strength of 100 MPa for Type II, Class 1 indications. This suggests that CS is mechanically suitable for posterior single crowns under normal occlusal loads [19].

Recently, a novel 3D-printable material called Ceramic Crown (CC) was introduced to the market. CC is manufactured additively using digital press stereolithography (DPS) technology, which employs hydrodynamic principles to move the material through the stereolithography process using a novel equipment (MIDAS, Sprintray, Los Angeles, CA, USA). Unlike digital light processing (DLP) printers, DPS replaces the resin vat and build platform with a single-use pressurized resin capsule. According to the manufacturer, this innovation allows for greater speed, accuracy, and ease in handling highly viscous materials. CC is indicated for partial- and full-coverage indirect restorations and is reported to exhibit mechanical properties including flexural strength > 200 MPa and elastic modulus > 15 GPa [20].

As no independent scientific evidence on the fracture resistance of this new CAD/CAM material is currently available, the present in vitro study aimed to compare its fracture resistance with that of other materials. The null hypothesis was that there would be no significant differences in the fracture resistance values of restorations fabricated with the different CAD/CAM materials.

## 2. Materials and Methods

### 2.1. Sample Preparation

A prefabricated maxillary typodont was used with a preparation for an indirect full-coverage restoration on the right maxillary first molar, featuring the following characteristics: 2 mm occlusal reduction, 1.5 mm axial reduction, chamfer finish line, axial walls with six degrees of taper, and rounded angles. Burs with different grit sizes were employed: green ring (100–120 μm), red ring (30 μm), and yellow ring (20 μm) (Master Prep, AXIS Dental; Crissier, Switzerland). Based on these preparations, indirect full-coverage restorations were digitally designed and divided into four groups (n = 40), corresponding to the following materials: VE (n = 10), EM (n = 10), CS (n = 10), and CC (n = 10). Each group consisted of 10 specimens, for a total of 40 samples. The characteristics of the materials used in this study are described in Table 1 [17,18,19,20,21].

The prefabricated model was digitized using an intraoral scanner (PrimeScan; Dentsply Sirona, Bensheim, Germany), and a full-coverage indirect restoration was designed with CAD software (InLab SW 22.3; Dentsply Sirona, Charlotte, NC, USA). The CAD file of the typodont was exported to a CAM system to mill (MCX5; Dentsply Sirona, USA) a master die (Vanilla Speed Wax Blank; AP Aesthetic-Press, New Port Richey, FL, USA). The wax-milled die was replicated in cast metal using the lost-wax technique.

**Figure 1:**
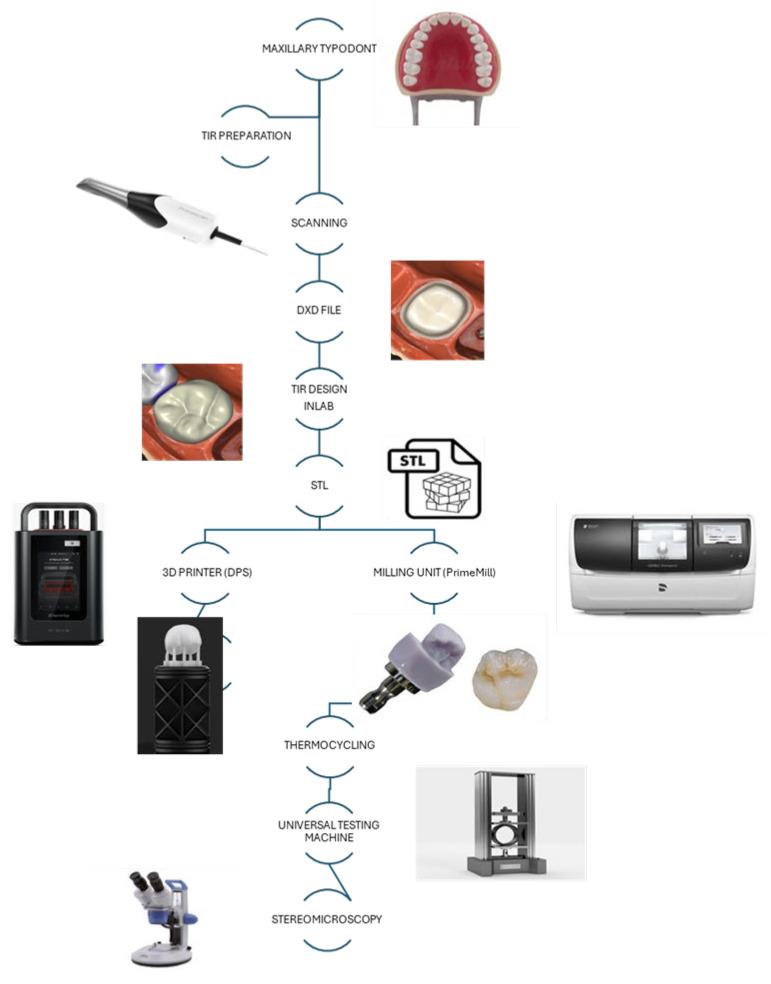
Workflow diagram of the experimental protocol: specimen preparation, intraoral scanning, digital design, STL file export, fabrication by subtractive milling or additive 3D printing, post-processing, thermocycling (for all groups), and evaluation procedures including stereomicroscopic analysis and fracture resistance testing.

The restoration design was transferred to a compact milling unit (PrimeMill; Dentsply Sirona, USA) in fine mode to obtain milled samples corresponding to the subtractive materialization groups. The 3D-printed group was fabricated using a 3D printer based on digital press stereolithography (DPS) (MIDAS, Sprintray; CA, USA) with a layer thickness of 100 µm, which is the only setting available for this system. Subsequently, the printed specimens underwent a washing and drying phase in an automated system (Procure, Sprintray; CA, USA), followed by post-curing in a specific unit (Nanocure, Sprintray; CA, USA) for three minutes, according to the manufacturer’s recommendations (Figure 1).

### 2.2. Sample Aging and Fracture Measurements

To simulate aging, specimens were subjected to thermocycling for 5000 thermal cycles between 5 °C and 55 °C, with a mean temperature of 37 °C, in distilled water (immersion time: 25 s; dwell time: 10 s) in a computerized thermocycling unit (Thermocycler OMC 300TS, Odeme Dental Research; Luzerna, Brazil). The specimens were then dried and inspected for cracks, chipping, or fractures following this phase.

Fracture resistance of the full-coverage restorations was evaluated under ambient conditions using a universal testing machine (Shimadzu AGS-X series, Shimadzu, Tokyo, Japan), with the master metal die fixed to the testing platform. The fit and seating of the specimens were verified using a 50 μm explorer. A load was applied at the center of the occlusal surface using a 3 mm diameter hardened steel spherical indenter at a crosshead speed of 0.5 mm/min until failure. The maximum fracture load was recorded in newtons (N). Finally, selected specimens were randomly observed under a stereomicroscope (MU1403; AmScope, United Scope, LLC, Irvine, CA, USA) at 40× magnification to identify failure mode, origin, and crack propagation direction.

### 2.3. Statistical Analysis

Statistical analyses were performed using SPSS software (v27.0; IBM Corp, Armonk, NY, USA). The Shapiro–Wilk test was used to assess the assumption of normality and Levene’s test to evaluate homoscedasticity. Due to normal distribution but lack of homogeneity of variances, Welch’s corrected parametric ANOVA was applied, followed by Games–Howell post hoc tests for multiple comparisons. Statistical significance was set at *p*-value < 0.05.

## 3. Results

### 3.1. Fracture Resistance

The EM group exhibited the highest fracture resistance, with a mean of 440.49 N, significantly higher than the other materials. In contrast, VE showed the lowest resistance, with a mean of 173.82 N, differing from the remaining groups. CS and CC presented intermediate values (265.49 N and 306.76 N, respectively). The coefficient of variation was highest for CC (23.8%), indicating greater heterogeneity in its results, whereas CS had the lowest variability (7.0%), suggesting greater uniformity in its behavior. The results showed statistically significant differences in fracture resistance among the four materials evaluated (*p*-value < 0.001). Multiple comparison tests revealed that the fracture resistance of EM and VE differed significantly from all other groups, while CS and CC did not show significant differences from each other. These findings indicate that EM is the material with the highest fracture resistance for indirect full-coverage restorations, whereas VE may be less suitable in terms of mechanical strength, and CS and CC offer similar intermediate fracture resistance (Table 2).

**Figure 2:**
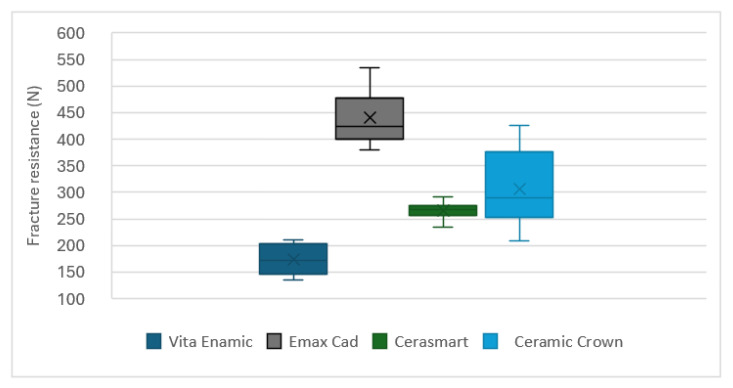
A boxplot diagram showing fracture resistance (N) for the four tested materials: Vita Enamic (VE), IPS e.max CAD (EM), Cerasmart (CS), and Ceramic Crown (CC). The plot displays median, interquartile range, minimum and maximum values, and mean for each group.

According to the boxplot diagram, EM exhibited the highest fracture resistance with a broad range, with values approximately between 375 N and 534 N and relatively low dispersion. VE was the least resistant and most homogeneous in behavior, with values concentrated approximately between 134 N and 210 N. CS presented a more compact distribution, with values between 235 N and 291 N, suggesting greater uniformity, while CC exhibited greater dispersion compared to the other materials, with values ranging from 207 N to 416 N (Figure 2).

### 3.2. Fracture Patterns

**Figure 3:**
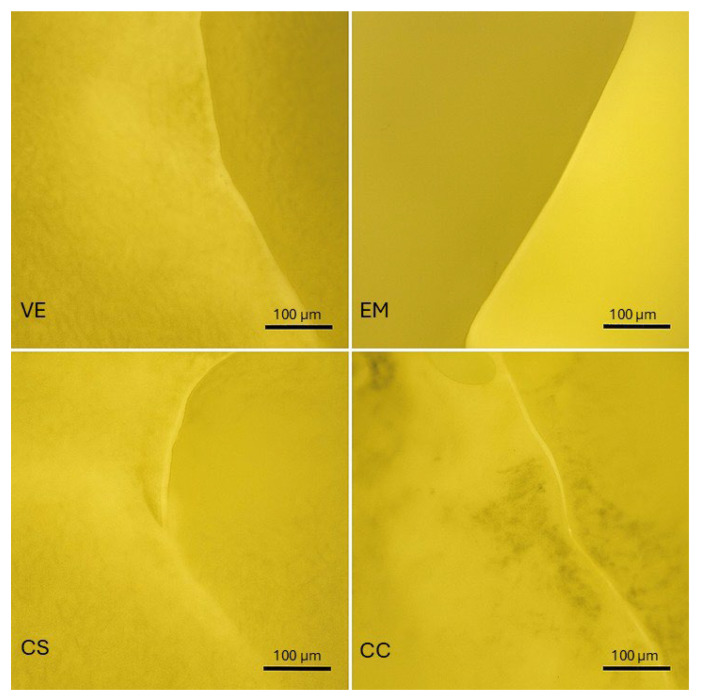
Stereomicroscopic images showing representative fracture patterns and crack propagation in each material group: VE, EM, CS, and CC. Characteristic defects and fracture lines are indicated for visual comparison.

The fracture micrographs in Figure 3 correspond to the average values of the compression load analyzed. The EM (monophasic lithium disilicate) fractured with smooth, straight planes, showing a brittle collapse with no appreciable energy dispersion. On the other hand, both VE (86% feldspar + 14% acrylate) and CS (71% ceramic nanoparticles + 29% polymer matrix) presented sinuous fracture profiles. These crack deviations suggest energy dissipation mechanisms in which the polymer phase slows down the crack propagation. CC (resin with 70% ceramic filler, DPS) presented an irregular fracture front with marked changes in direction. Layer-by-layer deposition introduces anisotropies and interlaminar discontinuities that could act as crack initiators. This profile with changes in direction explains its intermediate compressive strength compared to that of a purely feldspathic composite and that of the homogenized monolithic material. Controlling heterogeneities during processing is important for optimizing the mechanical reliability of indirect restorations.

## 4. Discussion

This in vitro study evaluated the fracture resistance in newtons of four CAD/CAM restorative materials, three of them representing subtractive manufacturing technologies and widely used in the market: a feldspathic ceramic with lithium disilicate, EM (mean: 440.49 N); a hybrid material, VE (173.82 N); a polymer-based material, CS (265.49 N); and CC (306.76 N), a novel material recently introduced alongside a new 3D printing technology: digital press stereolithography. As a result of the tests, EM exhibited the highest fracture resistance and VE the lowest, while CS and CC showed intermediate values.

The null hypothesis was rejected based on the results, which showed statistically significant differences among the materials tested. Due to the recent introduction of this 3D-printed resin material, there are currently no independent studies comparing its fracture resistance to that of milled resinous materials. When evaluating new materials, it is essential to assess their mechanical properties, with fracture resistance being a key parameter according to ISO 6872:2024 [22]. Other relevant properties may include flexural strength, elastic modulus, surface hardness, degree of conversion, and resistance to aging. However, such data for the new 3D-printed resin are not yet available in the literature. In this study, fracture resistance was selected as the primary variable, considering its clinical relevance under functional conditions where restorations must endure repetitive occlusal loads, thermal cycling, and humidity. These conditions were simulated in our protocol using thermomechanical aging [23].

CAM technology can be either subtractive or additive, and both have advantages and disadvantages. Milling systems offer high-dimensional accuracy and precision and good marginal and internal fit, and they are free from curing-related deformation. However, they present disadvantages such as high acquisition and maintenance costs, material waste, and limitations in achieving complex shapes and fine details. In contrast, 3D printing systems offer lower production costs, reduced material waste, faster production of multi-unit restorations, and the ability to fabricate complex shapes and customized designs. The disadvantages include lower material longevity, reduced dimensional accuracy, and potential deformation during the curing process [24].

The specimens fabricated from printed resin CC (306.76 N) exhibited similar fracture resistance to milled resin CS (265.49 N), which could be attributed to several factors such as filler content—both materials having approximately 70–71% filler—and differences in fabrication method. CS, being a subtractively manufactured material, is subject to machinability, defined as the material’s interaction with milling, which can variably cause undesired chipping [25]; in this context, CS exhibited intermediate machinability compared to EM and VE. On the other hand, it has been shown that in 3D-printed resins, higher filler content may impair resin flow during printer platform movements, increasing the risk of incorporating air bubbles, leading to porosity and negatively affecting mechanical properties [26]. However, CC specimens fabricated via 3D printing using hydrodynamic pressurization may have an advantage due to the pressurized delivery of the liquid material during printing.

As a new polymer-based material entering the market together with a new printing technology, CC seeks to improve upon these disadvantages by developing a polymeric material infiltrated with ceramic filler suitable for 3D printing, whereas such materials have so far been available only for milling systems. Although the exact composition of the material is not disclosed, the manufacturer reports it as a resin with approximately 60% ceramic filler by weight. The fracture resistance behavior observed in this study resembles that of the polymer-based CS material, which contains 71% filler nanoparticles (silica and barium glass) and 29% polymers such as Bis-MEPP, UDMA, and DMA [27]. This similarity suggests that the clinical applications of CC may be comparable to those of Cerasmart; however, additional studies using different variables are needed to better understand the mechanical behavior of this new material.

Fracture resistance is a critical parameter when evaluating the mechanical performance of restorative materials. When comparing fracture resistance among materials, EM showed the highest value (440.49 N), followed by CC (284.34 N), CS (265.49 N), and VE (173.82 N). The results of the present study do not align with those of Se-Jun An et al., in which VE and CS exhibited similar values [28]. This discrepancy could be explained by the composition of VE, a PICN (polymer-infiltrated ceramic network) material with a porous ceramic network (86%) infiltrated with acrylate polymer (14%), resulting in a potentially weak interface that acts as a fragile point, consistent with findings by Long Ling et al. [29] and Bankoglu et al., who recommended limiting VE use to the premolar region [30]. Furthermore, the fracture resistance values did not exceed the average reported occlusal forces in men (600–800 N) [31]; however, the lower values for CC, CS, and VE compared to EM suggest that caution should be exercised when using restorations fabricated from these three materials, regardless of manufacturing technique, in posterior regions.

The values recorded for EM (440.49 N) reflect its high fracture resistance. As a feldspathic ceramic with lithium disilicate, it offers a balance between strength and aesthetics, making it ideal for withstanding masticatory forces, and its high flexural strength (360–400 MPa) contributes to its durability [32]. However, lithium disilicate glass-ceramics have been shown to cause greater wear on opposing teeth due to their mechanical properties, in contrast to resin-ceramic materials, which are known to cause less antagonist wear [33].

To our knowledge, this is one of the first independent in vitro studies to evaluate both the DPS additive manufacturing technology and the novel CC material. The findings of this investigation provide early evidence suggesting that CC may achieve mechanical performance comparable to established milled resin-based composites, while offering the potential production advantages associated with additive manufacturing.

Nevertheless, due to the novelty of this material and the absence of long-term clinical validation, these results should be interpreted with caution and serve as a foundation for future research. In this study, crowns were tested in a non-cemented condition to isolate the intrinsic mechanical behavior of each material, eliminating the influence of adhesive interfaces. As expected, this protocol resulted in lower absolute fracture resistance values compared to cemented crowns, with the 3D-printed CC group reaching a mean of approximately 306 N. These values remain within the range of reported functional forces for anterior and premolar regions under normal occlusal conditions. In addition, the observed coefficients of variation (CVs) ranged between 10% and 25%, which is consistent with previously published in vitro studies and reflects the inherent variability of mechanical testing in brittle and resin-based materials.

It is important to note that no adhesive protocol was employed in this test. The literature consistently shows that fracture resistance significantly increases when adhesive procedures are applied, as adhesion provides micromechanical retention to the substrate and enables the restoration and tooth to function as a unified structure that better distributes occlusal forces and minimizes stress concentration [7,34].

However, incorporating an adhesive procedure would introduce additional variables such as type of cement, cement layer thickness, polymerization shrinkage, and adhesive system used [35]. Previous studies have reported that cement layer thicknesses between 50 and 100 µm provide better mechanical performance, while thicker layers (450–500 µm) result in diminished performance due to polymerization shrinkage stresses [36]. Regarding adhesive systems, their importance is heightened in indirect restorations as they involve multiple adhesive interfaces (at the tooth surface and at the restoration surface). Three-step and two-step adhesive systems are preferred over self-adhesive or one-step systems, as they provide higher bond strength and consequently greater fracture resistance [37]. Therefore, the fracture resistance values obtained in the present study reflect the intrinsic resistance of the material, which is particularly relevant since no data are currently available regarding the mechanical properties of the printable resin evaluated here.

According to the manufacturer, milling time for VE in standard mode is 9:07 min and 5:13 min in fast mode [17]. For CS, milling times range from 8 to 12 min in standard mode and 4 to 7 min in fast mode [19]. EM requires approximately 14:58 min in standard mode and 12:14 min in fast mode [38] per restoration. However, milling time is directly influenced by factors such as the type of milling unit, restoration complexity, and milling machine settings [39]. In this study, a milling unit (PrimeMill; Dentsply Sirona, USA) in fine mode was used, allowing for a high level of detail, particularly in occlusal fissures and interproximal areas, optimizing the precision of full-coverage restorations. On the other hand, additive manufacturing of CC, thanks to DPS printing technology and the use of disposable pressurized cartridges, allowed for the simultaneous printing of three crowns in 10 min, with an additional 3 min post-curing cycle. In total, three crowns were fabricated in 13 min, representing a clear time efficiency advantage compared to subtractive manufacturing methods: while milling one VE, EM, or CS crown, it is possible to print three CC crowns, offering significant clinical time savings in chairside environments. In terms of cost–benefit, milled hybrid materials VE and CS may be more expensive compared to CC. Nevertheless, the fracture resistance results in this study showed that VE had the lowest values, while CC and CS demonstrated intermediate performance. It is important to note that CC is a novel material and requires comprehensive clinical evaluation before its indications can be definitively validated. Finally, although EM is not a hybrid material, it was included in this analysis due to its widespread acceptance and clinical use, enabling relevant comparisons with alternative hybrid materials [40].

This study applied cyclic fatigue aging to obtain relevant results; however, it has limitations as an in vitro design. Moreover, a metallic die was used instead of natural dental abutments, and differences in elastic modulus may influence fracture resistance results. Nonetheless, using abutments with similar elastic moduli could mask the true performance of the materials or result in catastrophic fractures attributable to the die material rather than the tested material. The limited sample size could also account for the variability observed in the CC group. Additionally, fracture resistance may be influenced by the adhesive technique; therefore, future studies should include fractographic analyses that incorporate the effect of cementation.

## 5. Conclusions

EM restorations exhibited the highest fracture resistance, making them ideal for high-load areas and full-coverage restorations. In contrast, the hybrid ceramic (VE) showed the lowest resistance, suggesting it may be more appropriate for low-load areas or partial restorations. The polymer-based materials, milled (CS) and 3D-printed (CC), demonstrated intermediate fracture resistance values, with no statistically significant differences between them. These results suggest that DPS additive technology can match the mechanical performance of subtractive techniques while offering production advantages. As this study represents one of the first analyses of the DPS technology and the Ceramic Crown material, the findings provide valuable initial insights into its mechanical behavior. However, further research is warranted to validate its performance in clinical settings before widespread adoption.

## Figures and Tables

**Table 1:** A detailed description of the materials evaluated in this study, specifying manufacturing method (subtractive or additive), flexural modulus (FM) [21], commercial name, abbreviation, manufacturer, composition, and corresponding lot code.

GROUP	MATERIAL	FM	ABRV.	MANUFACTURER	COMPOSITION	LOT CODE
**1.** **Subtractive**	VITA Enamic	23 GPa	VE	Vita Zahnfabrik; Bad Säckingen, Alemania	Feldspathic ceramic 86% and acrylate polymer 14% [17].	219250
**2.** **Subtractive**	IPS E.max CAD	52 GPa	EM	Ivoclar Vivadent; Schaan, Liechtenstein, Alemania	Feldspathic ceramic reinforced with lithium disilicate [18].	YBC324
**3.** **Subtractive**	Cerasmart (Polymeric)	25 GPa	CS	GC Corporation; Tokyo, Japan	Silica nanoparticles (20 nm) and barium glass (300 nm), 71 wt%, Bis-MEPP, UDMA, and DMA polymers, 29 wt% [19].	2401171
**4.** **Additive**	Ceramic Crown (Polymeric)	70 GPa	CC	Sprintray; CA, USA	Resin with 70% ceramic filler [20].	M24J012

**Table 2:** Fracture resistance (N) of the tested materials: mean, standard deviation (SD), minimum, and maximum values.

Material	Mean (N)	Standard Deviation	Minimum (N)	Maximum (N)
VE	173	30	134.34	209.67
EM	440	56	377.50	534.02
CS	265	18	235.13	291.32
CC	306	73	207.97	426.58

## Data Availability

No new data were created or analyzed in this study. Data sharing is not applicable to this article.

## Abbreviations

The following abbreviations are used in this manuscript:

DPS	Digital Press Stereolithography
CAD/CAM	Computer-aided design/computer-aided manufacturing
EM	E.MAX CAD
VE	Vita Enamic
CS	Cerasmart
CC	Ceramic Crown
